# Pulmonary Actinomycosis: A Hidden Threat with Clinical Impact

**DOI:** 10.3390/arm94030033

**Published:** 2026-05-18

**Authors:** Raffaella Griffo, Jasmin K. Jasuja, Benedikt Niedermaier, Sabine Wege, Janina Shala, Henrike Deissner, Lena Brendel, Romina M. Rösch, Florian Eichhorn, Michael Allgäuer, Elizabeth Tong, Cosmas Wimmer, Martin E. Eichhorn, Hauke Winter, Laura V. Klotz

**Affiliations:** 1 Thoraxklinik, Department of Thoracic Surgery, Heidelberg University Hospital, 69126 Heidelberg, Germany; 2 Department for Infectious Diseases, Heidelberg University Hospital, 69126 Heidelberg, Germany; 3 Institute for Microbiology, Virology and Hygiene, University Medical Center Hamburg-Eppendorf, 20251 Hamburg, Germany; 4 Division of Systems Biology of Signal Transduction, German Cancer Research Center (DKFZ), 69120 Heidelberg, Germany; 5 Translation Lung Research Center (TLRC), German Center for Lung Research (DZL), 69120 Heidelberg, Germany; 6 Thoraxklinik, Department of Pneumology, Heidelberg University Hospital, 69126 Heidelberg, Germany; 7 Department of Pathology, Heidelberg University Hospital, 69120 Heidelberg, Germany; 8 Thoraxklinik, Department of Diagnostic and Interventional Radiology with Nuclear Medicine, Heidelberg University Hospital, 69126 Heidelberg, Germany; 9 Department of Diagnostic and Interventional Radiology (DIR), Heidelberg University Hospital, 69126 Heidelberg, Germany

**Keywords:** pulmonary actinomycosis, thoracic infection, lung mass, thoracic surgery, diagnostic delay

## Abstract

**Highlights:**

**What are the main findings?**

- Pulmonary actinomycosis is frequently misdiagnosed as lung malignancy, leading to substantial diagnostic delay.
- Bronchoalveolar lavage combined with microbiological testing provides the highest diagnostic yield compared to other modalities.
- *Actinomyces odontolyticus* was the most identified species, differing from previous reports and suggesting regional microbiological variability.
- Surgical intervention plays a key diagnostic and therapeutic role, achieving complete disease eradication in selected patients.

**What are the implications of the main findings?**

- Increased clinical awareness is essential to include pulmonary actinomycosis in the differential diagnosis of lung masses and avoid unnecessary delays.
- Early implementation of bronchoscopy with microbiological analysis may improve diagnostic accuracy and patient management.
- A multidisciplinary approach, including appropriate surgical indication and improved adherence to dental follow-up, may reduce recurrence and optimize outcomes.

**Abstract:**

Background: Pulmonary actinomycosis is a rare chronic infection that frequently mimics lung malignancy, often leading to delayed diagnosis due to its non-specific clinical and radiological presentation. Given the diagnostic challenges associated with this condition, the aim of this study was to evaluate the clinical presentation, diagnostic pathways, treatment strategies, and outcomes of patients diagnosed with pulmonary actinomycosis in a single center. Methods: We retrospectively reviewed patients diagnosed with pulmonary actinomycosis at our institution between January 2014 and December 2022. Diagnosis was established based on compatible clinical and radiological findings together with microbiological identification of *Actinomyces* by culture or polymerase chain reaction. Results: Twenty-two patients were included in the final analysis. The median age was 61.5 years and males were more frequently affected (59%). The median time from initial hospitalization to definitive diagnosis was 70 days. *Actinomyces odontolyticus* was the most frequently identified species. All patients received antibiotic therapy, with a median treatment duration of 45.5 days. Thirteen patients underwent surgical intervention, performed either for diagnostic purposes or for treatment of complications. Complete disease eradication through surgical management was achieved in six cases. During follow-up (median 24 months), overall survival at three years was 78%, with one death directly related to pulmonary actinomycosis. Conclusions: Pulmonary actinomycosis remains a diagnostic challenge due to its non-specific clinical presentation and low microbiological yield. Early clinical suspicion and a combined diagnostic approach including bronchoscopy and microbiological testing are essential for timely diagnosis. Surgical intervention may play an important diagnostic and therapeutic role in selected patients.

## 1. Introduction

Pulmonary actinomycosis (PA) is a rare, chronic infection caused by *Actinomyces species plurima* (spp). Diagnosis is challenging due to its ability to mimic malignancy both clinically and radiologically [1]. The incidence of PA has markedly declined in developed countries following the widespread use of antibiotics, making it a less familiar entity in clinical practice.

Several studies have reported that up to 25% of PA cases are initially misdiagnosed as lung cancer because of overlapping clinical and radiological features [2,3]. The disease typically presents with non-specific respiratory symptoms such as chronic cough, hemoptysis, and chest pain, which are also common in other pulmonary disorders [4,5,6]. Recent literature emphasizes the impact of imaging in differentiating pulmonary actinomycosis from other diseases. Radiological findings can further obscure diagnosis, often resembling those seen in lung malignancies or other chronic infections [7,8]. PA may present with increased uptake on positron emission tomography (PET), further mimicking malignant disease [9]. The presence of sulfur granules, a hallmark of actinomycosis, can be seen in other inflammatory conditions, complicating the diagnostic process [10].

Demographics of patients with PA show a male predominance, with risk factors including poor oral hygiene, smoking, and underlying lung disease [6,11]. Although the disease can occur in otherwise healthy individuals, it is often associated with immunocompromised conditions [12].

Treatment of PA typically involves prolonged courses of high-dose intravenous penicillin, which is effective against the bacteria that cause the disease. However, the treatment regimen can be complicated by fibrous lesions and poor vascularization in the affected areas, requiring careful management [13]. Antibiotics are usually effective, but delays in diagnosis can lead to clinically significant complications such as hemoptysis or progression of the disease [5].

The existing literature highlights the need for increased awareness and improved diagnostic strategies for PA, particularly in patients with respiratory symptoms that may be misattributed to cancer or other infections. Imaging techniques and a clinical history and microbiologic evaluation are essential for an accurate diagnosis and effective treatment.

The aim of this study was to retrospectively analyze the clinical presentation, diagnostic pathways, treatment strategies, and outcomes of patients with pulmonary actinomycosis treated at our institution.

## 2. Methods

### 2.1. Study Population

Patients were retrospectively selected from a prospectively maintained database at Thoraxklinik Heidelberg between January 2014 and December 2022. Diagnosis of pulmonary actinomycosis was based on compatible clinical and radiological findings together with microbiological identification of *Actinomyces* spp. by culture or polymerase chain reaction (PCR). Histological findings were considered supportive but were not sufficient for diagnosis in the absence of microbiological confirmation. Patients with other primary conditions in which *Actinomyces* spp. were incidentally detected, without evidence of PA, were excluded from the study.

Clinical manifestation was defined as the presence of persistent, non-specific respiratory symptoms. Radiological evidence was defined as the presence of lesions in the mediastinum, lungs, or pleural cavity detected on computed tomography (CT) imaging. The clinical and radiological manifestation of the disease, the time interval between initial hospitalization and definitive diagnosis, and the diagnostic procedures required for diagnosis were reviewed. Finally, we conducted an analysis of the therapeutic approach, evaluating the selected antibiotic treatment and its duration. The need for surgical intervention was also assessed, including the purpose of the procedure, whether diagnostic or therapeutic. Any complications or patient deaths during the follow-up period were recorded.

This study was conducted in accordance with the principles of the Declaration of Helsinki for ethics in medical research. The collection and analysis of data from patients’ medical records were approved by the institutional ethics committee of Heidelberg University Hospital, waiving the requirement for individual informed consent (No. S-174/2019).

### 2.2. Follow Up

Patients were monitored after completing therapy through a follow-up protocol that included at least one clinical and radiological assessment. As part of the radiological evaluation, a chest CT scan was performed to assess disease remission. In addition, a dental examination was conducted to exclude the presence of a potentially remaining odontogenic focus. Where disease persistence or progression was suspected, further microbiological investigations were carried out to detect *Actinomyces* spp. Patients who lived far from our hospital underwent a follow-up visit with their primary care physician and were contacted by phone by our team. Follow-up data was completed in May 2025.

### 2.3. Statistical Analysis

Statistical analysis was performed using Prism (version 10.6.0, Graphpad Software Inc., Boston, MA, USA). A descriptive analysis was conducted to summarize demographic, clinical, and pathological characteristics. Continuous variables were reported as medians with interquartile ranges, while categorical variables were presented as counts and percentages. Overall survival, defined as the time between final diagnosis and death, was analyzed using the Kaplan–Meier method. A *p*-value of less than 0.05 was considered statistically significant.

## 3. Results

### 3.1. Patient Characteristics and Comorbidities

**Figure 1:**
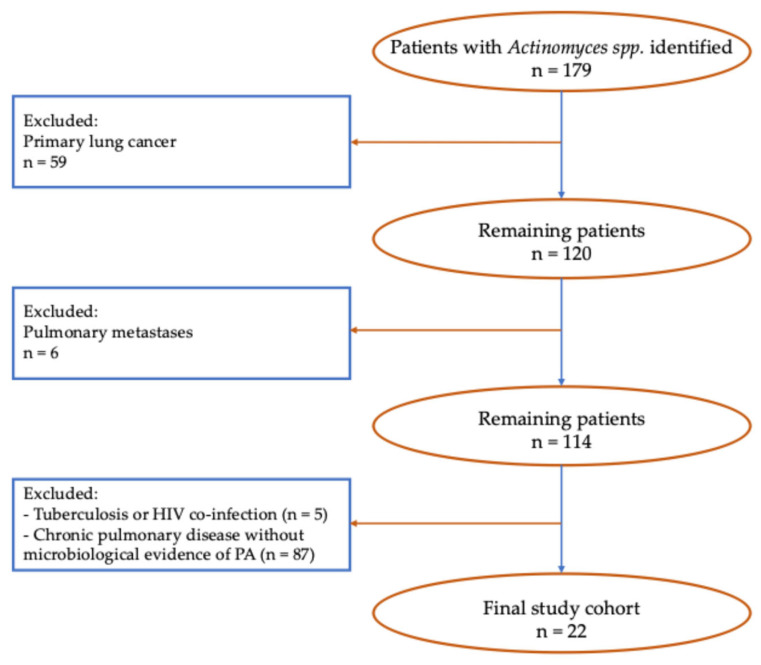
Flowchart of the patient selection process for pulmonary actinomycosis (PA).

The patient selection process is illustrated in Figure 1. Among the 179 patients diagnosed with *Actinomyces* spp. between January 2014 and December 2022, 59 patients with primary lung cancer were excluded. Of the remaining 120 patients, 6 with pulmonary metastases were excluded, resulting in a cohort of 114 patients. Additional exclusions included patients with co-infection by tuberculosis or human immunodeficiency virus (HIV) (n = 5) and those with chronic pulmonary diseases without histological or microbiological evidence of actinomycosis (n = 87). After applying these criteria, 22 patients with confirmed pulmonary actinomycosis were included in the final analysis.

Their demographic characteristics and comorbidities are detailed in Table 1. The median age was 61.5 years (interquartile range (IQR) 49.5–68.0 years). Males were more frequently affected (59% versus 41%) and median body mass index (BMI) was 23 kg/m^2^ (IQR 19.1–27.8 kg/m^2^). Blood gas analysis was performed at initial presentation using capillary samples obtained under ambient air conditions. The median PaO_2_ was 73.5 mmHg (IQR 66.5–75.7), suggesting mild hypoxemia in a proportion of patients. With regard to smoking status, eight patients (36.4%) were never smokers, while seven (31.8%) were former smokers and seven (31.8%) were current smokers. Data on smoking exposure were available for 21 patients, with a median of 10 pack-years (IQR 0–30). Half of the patients exhibited an excellent physical performance status, as indicated by an Eastern Cooperative Oncology Group (ECOG) score of 0. Concomitant immunosuppression was found in 11 patients, including alcohol abuse (2 patients), drug use (1 patient), and prolonged systemic corticosteroid therapy (6 patients). Among the 11 patients with documented pulmonary comorbidities, chronic obstructive pulmonary disease (COPD) was the most frequently observed condition. Cardiovascular comorbidities were present in 63.5% of patients. Only one patient was found to have a history of previous urothelial carcinoma. No other patients had associated oncological conditions (see Table 1 for details).

### 3.2. Clinical and Radiological Presentation

The most common presenting symptoms were influenza-like symptoms, reported in 14 patients (63.6%), including fever, malaise, and general fatigue. These were followed by hemoptysis (3 patients), dyspnea (3 patients), cough (2 patients), and dysphagia (1 patient), as detailed in Table 2.

Radiological findings showed pneumonic infiltrates with atelectasis in 7 patients. Diffuse lymphadenopathy was observed in 5 cases, pulmonary consolidations in 4, and pulmonary abscesses in 3 patients. Pleural empyema, which represents a more advanced disease stage, was identified in only 3 patients.

The median time from initial hospitalization to definitive diagnosis was 70 days. During this period, most patients were discharged and subsequently readmitted for further diagnostic evaluation, symptom progression, or treatment adjustment. The median number of hospital admissions before establishing the diagnosis was 3.5 (Table 2).

### 3.3. Diagnostic Methods

**Figure 2:**
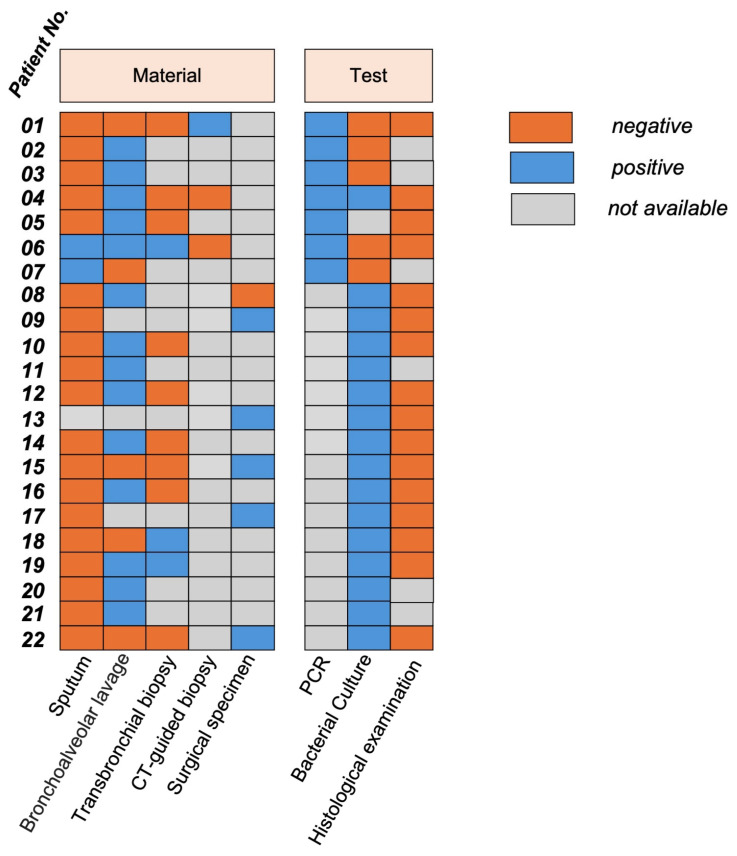
Diagnostic specimens and tests performed in the study cohort. Specimen types include sputum, bronchoalveolar lavage, transbronchial biopsy, CT-guided biopsy, and surgical specimens. Diagnostic methods comprise polymerase chain reaction (PCR), bacterial culture, and histological examination. Test results are indicated as positive, negative, or not available for each patient.

A uniform diagnostic sequence was not applied, as the diagnostic approach was guided by the individual clinical presentation. The diagnostic specimens and methods used in the cohort are summarized in Figure 2, and the diagnostic yield of the different modalities is presented in Table 3.

Sputum analysis was performed in 21 patients and was positive in 2 cases (9.5%), while 19 patients tested negative (90.5%). Bronchoalveolar lavage (BAL), performed via bronchoscopy, was conducted in 20 patients and yielded positive results in 15 cases (75.0%) and negative results in 5 cases (25.0%). Transbronchial biopsy was performed in 12 patients, yielding 3 positive (25.0%) and 9 negative results (75.0%). CT-guided biopsy was performed in 3 patients, yielding 1 positive result (33.3%) and 2 negative results (66.7%). In five patients, the diagnosis was ultimately established from surgical specimens.

PCR testing was performed in seven patients and yielded positive results in all cases. Microbiological culture was conducted in 21 patients, yielding 16 positive results (76.2%). The identification of *Actinomyces* spp. was based on both microbiological culture and PCR analysis. In several cases, *Actinomyces* was detected by PCR despite negative or inconclusive culture results, which explains the discrepancy between the number of cultures performed and the total number of identified strains. Histological examination was performed in 16 patients and did not identify *Actinomyces* spp. in any case. However, histological findings were consistent with chronic inflammation and supported the clinical suspicion of infection.

### 3.4. Treatment and Clinical Outcome

**Figure 3:**
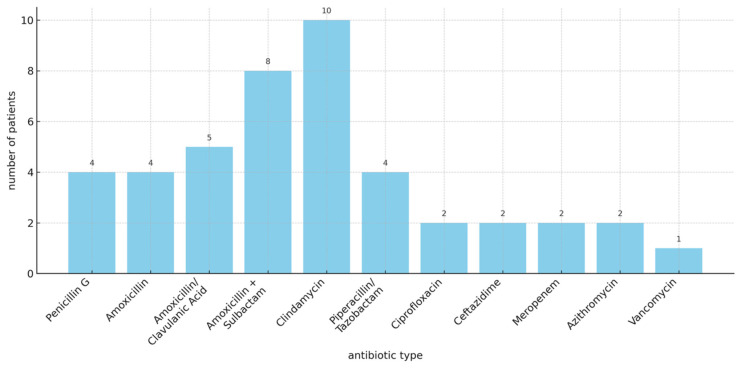
Distribution of antibiotic therapies administered in the study cohort. Patients frequently received multiple antibiotic agents, either sequentially or in combination, depending on clinical response and diagnostic findings. The figure reflects the overall use of antibiotics rather than a standardized treatment sequence.

*Actinomyces odontolyticus* was the most commonly identified species, followed by *Actinomyces meyeri* in three patients and three additional cases involving unspecified *Actinomyces* species (see Table 4). All patients received antibiotic therapy. Figure 3 summarizes the antibiotic regimens administered to patients from symptom onset until disease resolution. Several patients received multiple antibiotic regimens during the course of treatment. Clindamycin was prescribed in ten cases, followed by amoxicillin/sulbactam in eight cases. Amoxicillin/clavulanic acid was used in five patients. Penicillin G, amoxicillin, and piperacillin/tazobactam were each administered in four cases, respectively. The median duration of antibiotic therapy administered was 45.5 days (IQR 40.2–50.7) as shown in Table 4.

A total of 13 surgical procedures were performed within the cohort. In one case, a diagnostic thoracoscopy with pleural biopsy was carried out. Four patients underwent combined diagnostic and therapeutic procedures, including three thoracoscopic evacuations for empyema and one thoracotomy with lobectomy for extensive lung destruction. Several patients required therapeutic procedures only. In six cases, complete eradication of the disease was achieved.

Complications related to previous surgery included one case of recurrent empyema with wound dehiscence and one case of recurrent empyema after lobectomy. Other complications observed in the entire cohort included three cases of respiratory failure requiring oxygen therapy, one case of heart failure, and two cases of sepsis.

Patients who underwent surgical intervention generally presented with more advanced disease or complications such as empyema, whereas patients managed with antibiotic therapy alone tended to have less severe clinical presentations. Due to the limited sample size, no formal comparative analysis between surgical and non-surgical management was performed.

Four deaths occurred during the follow-up period, only one was attributed to the disease (see Table 4).

### 3.5. Long-Term Follow-Up and Survival Analysis

Among the 17 followed patients, four deaths were recorded: three were due to causes unrelated to pulmonary actinomycosis, while one patient died in the postoperative period due to complications directly associated with the disease.

Follow-up data was updated through May 2025. All 17 patients underwent at least one clinical evaluation including a chest CT scan during the follow-up period. Five patients underwent repeated microbiological testing on bronchial secretions with culture, and only one showed persistent positivity for *Actinomyces* spp.

All patients were advised to undergo a dental evaluation as part of the management strategy, but only eight complied. In two patients, a dental infectious focus was identified and removed.

**Figure 4:**
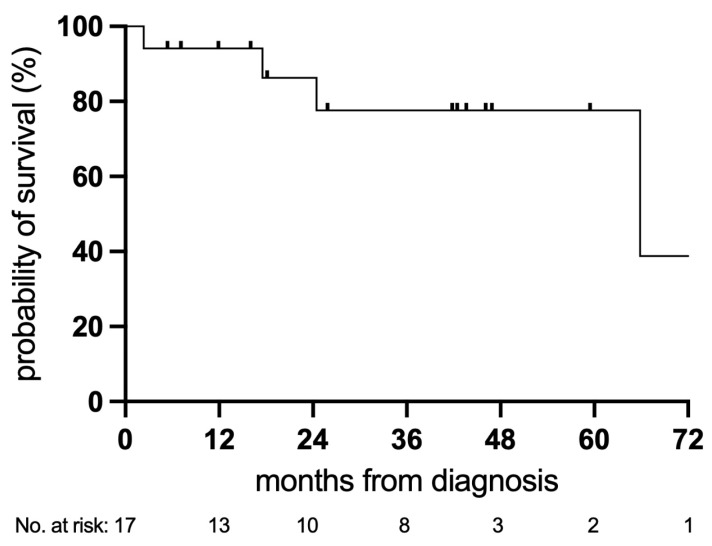
Kaplan–Meier survival curve illustrating overall survival from diagnosis in the study cohort (n = 17). Three-year overall survival was 78% (95% CI: 44.7–92.4%). Numbers at risk are shown below the x-axis, indicating the number of patients remaining under observation at each time point. Interpretation of later time points is limited by the small number of patients remaining under observation.

The Kaplan–Meier survival curve illustrates overall survival from diagnosis in the study cohort (n = 17, Figure 4). Most deaths occurred within the first 12 months. Three-year overall survival was 78% (95% CI: 44.7–92.4%). Interpretation of later time points is limited by the small number of patients remaining at risk.

## 4. Discussion

Pulmonary actinomycosis, a rare infection caused by *Actinomyces* species, is documented worldwide. Since the introduction of antibiotic therapy, its incidence has markedly declined in developed countries. Despite its decreasing incidence, pulmonary actinomycosis remains a diagnostic challenge due to limited clinical familiarity [14]. To provide additional evidence, we thoroughly analyzed our single-center experience with pulmonary actinomycosis, including data on patient characteristics, diagnostic pathways, and treatment. The diagnosis was often delayed due to the rarity of the pulmonary disease.

In our cohort, the median age at presentation was higher than in recent Asian studies, which reported a peak incidence in the fifth decade [15,16,17]. This difference may reflect demographic and healthcare-related differences. Consistent with previous reports, we observed a predominance of PA in males and in patients with immunosuppression. While chronic pulmonary conditions are recognized as potential risk factors for PA, this association has thus far been supported only by small case series [18]. Notably half of the patients in the present study were found to have an underlying chronic pulmonary comorbidity.

Pulmonary actinomycosis has been described by Boynova et al. as a ‘forgotten disease’ [14]. Its rarity often causes it to be omitted from the differential diagnosis, which significantly delays identification and appropriate management [19]. As reported by Boot et al., the correct initial diagnosis is made in only 4% of cases due to its non-specific clinical and radiological features. These findings are also supported by the results of the present study. Pulmonary actinomycosis was not initially suspected in any of the cases. The presence of a solid mass, either alone or with cavitation, was seen in 32% of patients on radiological imaging. In such cases, neoplastic disease is part of the differential diagnosis [20]. These presentations are often misleading. When a patient exhibits no overt signs of infection, and malignancy is suspected, a biopsy is not usually the initial approach. Instead, further staging investigations, such as a PET scan, are typically pursued first. In clinical practice, completing the staging process, reviewing the imaging results, and determining the next diagnostic step requires time [21]. This process takes at least two weeks, which delays the start of treatment for patients with active pulmonary actinomycosis. In cases where a biopsy is performed early, but there is no clinical suspicion of an infectious etiology, bronchoalveolar lavage with associated PCR analysis is often not conducted. As demonstrated by our data, consistent with findings in the literature, this combined approach represents the most accurate diagnostic method for confirming PA.

Another critical aspect of the disease is the difficulty of identifying the causative bacterium. Even when the condition is suspected from the beginning, isolation of *Actinomyces* through microbiological or histological methods is not achieved immediately in most cases. The median diagnostic delay of 70 days in our study is consistent with other studies and bears significant consequences [18]. Using empirical antibiotics without microbiological confirmation, as seen in some of our patients and reported in the literature [16], can mask infection, increase false-negative results, and promote resistance. Delayed diagnosis also increases the risk of disease progression to advanced complications such as pleural empyema, fistulas, or extrathoracic spread [21].

Notably, in our cohort, *Actinomyces* spp. was not identified histologically in any case, and concordance between culture and PCR was uncommon. Most diagnoses relied on a single diagnostic modality. PCR testing showed a high detection rate; however, it was performed in a limited number of selected patients. These findings should therefore be interpreted with caution and do not allow definitive conclusions regarding diagnostic accuracy.

Histological analyses consistently demonstrated chronic infection, characterized by necrosis and granulomatous inflammation. However, direct identification of the causative microorganism was never achieved. These findings underscore the heterogeneous nature of the diagnostic approach and the necessity of including microbiological examinations of clinical specimens.

In our cohort, *Actinomyces odontolyticus* was the most frequently identified species, in contrast to previous reports describing *Actinomyces graevenitzii* as the predominant pathogen in pulmonary actinomycosis [22,23,24]. This discrepancy may reflect differences in local microbiological patterns, referral bias at a thoracic surgery center, or the polymicrobial environment of the oral cavity. Notably, compliance with recommended dental evaluation during follow-up was low. Given that the oral cavity is a common source of *Actinomyces* infection, inadequate identification and management of odontogenic foci may contribute to disease persistence or recurrence. These findings underscore the importance of interdisciplinary management and improved adherence to follow-up recommendations. In addition, ongoing changes in the microbiological taxonomy of the *Actinomyces* genus, including the reclassification of previously described species, as well as variability in species-level identification across laboratories, may further explain discrepancies observed between studies [25,26].

Therapeutic management of pulmonary actinomycosis with antibiotics remains challenging, due to the prolonged treatment duration required and the variability in clinical response. As this cohort demonstrates, most patients required multiple biopsies before a definitive diagnosis could be made. In select instances, antibiotic treatment was initiated based on clinical signs of infection, even in the absence of a confirmed diagnosis. This approach may first and foremost compromise the reliability of subsequent diagnostic tests and contribute to delayed diagnosis due to an increased risk of false-negative results. Moreover, the antibiotic therapy administered to these patients is often empirical, broad-spectrum, and of limited duration. Such a short and non-aggressive therapeutic regimen may contribute to the development of antibiotic resistance and consequently to disease persistence. When a rapid diagnosis was achieved, the lack of familiarity with the disease often became evident, leading to insufficiently aggressive and inadequately long therapeutic approaches. This significantly reduces the likelihood of completely eradicating the infection.

In our cohort, the median duration of antibiotic therapy was shorter than traditionally recommended in the literature, where prolonged treatment courses of several months are often advised. This discrepancy may reflect variability in real-world clinical practice, particularly in cases where diagnosis is delayed or uncertain. In addition, surgical intervention in selected patients may have contributed to shorter antibiotic regimens by enabling removal of infected tissue. However, given the limited sample size and follow-up, no definitive conclusions can be drawn regarding the optimal duration of therapy.

Another critical issue is the lack of standardized international guidelines for diagnosing and managing pulmonary actinomycosis. Consequently, current clinical practice largely relies on case reports, small retrospective series, and expert opinion rather than evidence-based protocols. This lack of consensus contributes to substantial variability in diagnostic strategies and therapeutic regimens, particularly regarding the choice, dosage, and duration of antibiotic therapy. Therefore, the development of clear, evidence-based guidelines is urgently needed to harmonize clinical practice, improve diagnostic accuracy, and optimize therapeutic outcomes for patients with this rare and often underdiagnosed infection [27].

Surgical intervention continues to play a key therapeutic role in selected cases of advanced pulmonary involvement, especially when complications such as abscesses or empyema are present. This is important for managing complications and for potentially discontinuing antibiotic therapy. In clinical practice, it is not uncommon to encounter patients who are unable to undergo prolonged antibiotic treatment due to poor compliance or intolerance. In such cases, surgery becomes nearly indispensable. This study underscores the pivotal role of surgery in diagnosis and treatment of the disease. Thirteen patients underwent surgery, six of whom achieved definitive cure through the procedure, enabling complete resection of the disease and successful completion of antibiotic therapy. Several retrospective studies have reported cure rates above 90% and favorable long-term outcomes, particularly in patients with persistent symptoms such as hemoptysis, or in those unresponsive to medical therapy alone [21,28].

The main limitations of this study include its retrospective, single-center design and the relatively small sample size, which limit the generalizability of the findings and preclude multivariable analyses to identify independent prognostic factors.

The rarity and non-specific clinical presentation of pulmonary actinomycosis make prospective studies based on initial clinical suspicion difficult to conduct. In addition, the heterogeneity of the diagnostic work-up, including variability in imaging modalities and microbiological sampling techniques, may have influenced the diagnostic yield. The small cohort size and limited number of events also restrict the robustness of survival analyses, which should therefore be interpreted as descriptive only. Furthermore, the follow-up period was not uniform, potentially leading to an underestimation of recurrences and complications. Given the heterogeneity of the cohort and the limited sample size, subgroup analyses were not performed, as they would not allow for meaningful or statistically robust conclusions. Future studies with larger cohorts are needed to further explore differences between treatment strategies and patient subgroups.

## 5. Conclusions

Pulmonary actinomycosis should be considered an uncommon but important differential diagnosis in patients presenting with persistent pulmonary lesions. When clinically suspected, diagnostic confirmation should include at least bronchoalveolar lavage, with PCR testing preferred to improve diagnostic accuracy. Early initiation of targeted antibiotic therapy is essential to prevent disease progression and complications.

Surgical intervention plays a pivotal role not only in achieving a definitive diagnosis, but also as a curative treatment option in advanced or refractory cases. Complete surgical resection can enable full eradication of the infection and reduce the need for prolonged antibiotic therapy.

A comprehensive understanding of this rare disease, together with heightened clinical awareness and the development of standardized diagnostic and therapeutic guidelines, is essential to ensure timely recognition, appropriate management, and improved patient outcomes.

## Figures and Tables

**Table 1:** Baseline characteristics and comorbidities of entire cohort.

	Number (%) or Median (Range)
Age in years	61.5 (49.5–68)
Gender	
Male	13 (59%)
Female	9 (41%)
BMI [kg/m^2^]	23 (19.1–27.8)
FEV1 [L]	2 (1.1–2.6)
PaO_2_ (mmHg)	73.5 (66.5–75.7)
PaCO_2_ (mmHg)	38 (36–39.7)
Smoking status	
Never smoker	8 (36.4%)
Former smoker	7 (31.8%)
Current smoker	7 (31.8%)
Pack-years	10 (0–30)
	[n = 21]
ECOG-Status	
ECOG 0	11 (50%)
ECOG 1	10 (45%)
ECOG 2	1 (5%)
Immunosuppression	
Alcohol Abuse	2 (9.1%)
Drug Use	1 (4.5%)
Prolonged immunosuppressive therapy	6 (27.3%)
Pulmonary comorbidities	
COPD	5 (22.7%)
Asthma	1 (4.5%)
Pulmonary fibrosis	1 (4.5%)
Silicosis	1 (4.5%)
Bronchiectasis	2 (9.1%)
History of tuberculosis	1 (4.5%)
Cardiovascular comorbidities	
Hypertension	3 (13.6%)
Coronary artery disease	3 (13.6%)
Atrial fibrillation	2 (9.1%)
History of congestive heart failure	3 (13.6%)
Diabetes mellitus	3 (13.6%)
Previous cancer-related comorbidity	
Urothelial carcinoma	1 (4.5%)
None	21 (95.5%)

**Table 2:** Clinical presentation, radiological features, and hospitalization data.

	Number (%) or Median (Range)
Symptoms	
Hemoptysis	3 (13.6%)
Cough	2 (9.1%)
Dyspnea	3 (13.6%)
Flu-like symptoms	14 (63.6%)
Dysphagia	1 (4.5%)
Radiological findings	
Consolidation	4 (18.2%)
Lymphadenopathy	5 (22.7%)
Consolidation with cavitation	3 (13.6%)
Pleural empyema	3 (13.6%)
Pneumonic infiltrates and atelectasis	7 (31.8%)
Hospitalization Data	
Median number of hospital admissions	3.5 (3.0–4.0)
Time from initial admission to definitive diagnosis (days)	70 (6.6–102.6)

**Table 3:** Diagnostic yield of different modalities.

Modality [n Tested]	Number (%)
Sputum [n = 21]	
- Positive	2 (9.5%)
- Negative	19 (90.5%)
Bronchoalveolar lavage [n = 20]	
- Positive	15 (75.0%)
- Negative	5 (25.0%)
Transbronchial biopsy [n = 12]	
- Positive	3 (25.0%)
- Negative	9 (75.0%)
CT-guided biopsy [n = 3]	
- Positive	1 (33.3%)
- Negative	2 (66.7%)
Surgical specimens [n = 5]	
- Positive	5 (100%)

**Table 4:** Identified *Actinomyces* species, therapeutic management, and complications.

	Number (%) or Median (Range)
Identified *Actinomyces* subtypes	
*Actinomyces odontolyticus*	12 (54.5%)
*Actinomyces gerencseriae*	1 (4.5%)
*Actinomyces israelii*	1 (4.5%)
*Actinomyces meyeri*	3 (13.6%)
*Actinomyces oris*	1 (4.5%)
*Actinomyces graevenitzii*	1 (4.5%)
*Actinomyces* species	3 (13.6%)
Median duration of antibiotic therapy (days)	45.5 (40.2–50.7)
Surgical procedures	
Diagnostic purpose	
- Thoracoscopy with pleural biopsy	1 (4.5%)
Diagnostic and therapeutic purpose	
- Thoracoscopy with pleural empyema evacuation	3 (13.6%)
- Thoracotomy with pulmonary lobectomy	1 (4.5%)
Therapeutic purpose	
- Thoracotomy with pulmonary lobectomy	1 (4.5%)
- Thoracotomy with cyst excision	1 (4.5%)
- Chest drain after biopsy-induced pneumothorax	1 (4.5%)
- Chest drain placement for tension pneumothorax	2 (9.1%)
- Tracheostomy for prolonged weaning	1 (4.5%)
Complications related to previous surgery	
- Recurrent empyema with wound dehiscence	1 (4.5%)
- Recurrent empyema after lobectomy	1 (4.5%)
Other complications	
Respiratory failure requiring oxygen therapy	3 (13.6%)
Heart failure	1 (4.5%)
Sepsis	2 (9.1%)
Death	4 (18.2%)
- Disease-related death	1 (4.5%)
- Non–disease-related death	3 (13.6%)

## Data Availability

The data presented in this study are available from the corresponding author upon reasonable request.
